# Unraveling the epigenetic code in cancer cell–tumor microenvironment crosstalk

**DOI:** 10.1002/1878-0261.70321

**Published:** 2026-08-25

**Authors:** Ji Hoon Park, Mi‐Young Kim

**Affiliations:** ^1^ Department of Biological Sciences Korea Advanced Institute of Science and Technology (KAIST) Daejeon Republic of Korea; ^2^ KAIST Institute for the BioCentury Cancer Metastasis Control Center Daejeon Republic of Korea

**Keywords:** cancer epigenetics, chromatin modifications, epigenetic drugs, epitranscriptomics, tumor microenvironment

## Abstract

Epigenetics plays a central role in cancer development and progression by governing the expression of genes involved in these processes. Accumulating evidence strongly indicates that these mechanisms not only alter cancer cell‐intrinsic properties but also mediate reciprocal interactions between cancer cells and the tumor microenvironment (TME). In the context of cancer cell‐intrinsic changes, epigenetic alterations contribute to cancer cell plasticity by regulating stemness, cell state transitions, and therapy resistance. In addition, epigenetic changes are a key driver of the establishment of a tumor‐supportive environment by modifying the states of TME components, including fibroblasts, macrophages, and myeloid‐derived suppressor cells. Epigenetic reprogramming in cancer cells and TME components is often induced by factors such as hypoxia and the secretion of exosomal long noncoding RNAs (lncRNAs), cytokines, and metabolites. In this review, we discuss various epigenetic mechanisms controlling cancer cell‐intrinsic states, cancer cell–TME crosstalk, current epigenetic therapies, and future research directions that may lead to the discovery of new biomarkers and the development of effective epigenetic anticancer therapeutics.

## Graphical abstract

Epigenetic regulation is a key driver of cancer development and progression. Diverse epigenetic alterations in cancer cells and components of the tumor microenvironment (TME) orchestrate their communication through multiple mechanisms. We discuss how the epigenetic code coordinates bidirectional cancer cell–TME crosstalk to promote cancer progression.

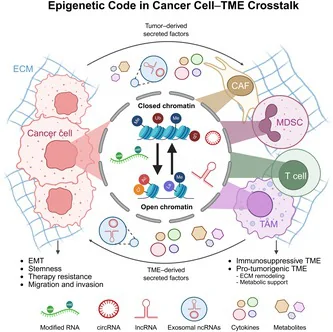

**Abbreviations**
2‐HG: 2‐hydroxyglutarate
apCAF: antigen‐presenting cancer‐associated fibroblast
BC: breast cancer
CAF: cancer‐associated fibroblast
ceRNA: competing endogenous RNA
circRNA: circular RNA
CRC: colorectal cancer
DNMT: DNA methyltransferase
ECM: extracellular matrix
EMT: epithelial–mesenchymal transition
ER^+^
: estrogen receptor‐positive
ESCC: esophageal squamous cell carcinoma
EZH2: enhancer of zeste homolog 2
GBM: glioblastoma
HDAC: histone deacetylase
iCAF: inflammatory cancer‐associated fibroblast
ICB: immune checkpoint blockade
IDH: isocitrate dehydrogenase
IFN: interferon
lncRNA: long noncoding RNA
m5C: 5‐methylcytosine
m6A: N6‐methyladenosine
MDSC: myeloid‐derived suppressor cell
meCAF: metabolic cancer‐associated fibroblast
MHC‐I: major histocompatibility complex class I
miRNA: microRNA
myCAF: myofibroblastic cancer‐associated fibroblast
ncRNA: noncoding RNA
NF‐κB: nuclear factor kappa B
NK cell: natural killer cell
NSCLC: non‐small‐cell lung cancer
OCC: ovarian cancer cell
PDAC: pancreatic ductal adenocarcinoma
PRC2: Polycomb repressive complex 2
ROS: reactive oxygen species
SAM: S‐adenosylmethionine
SASP: senescence‐associated secretory phenotype
TAM: tumor‐associated macrophage
TDG: thymine DNA glycosylase
TET: ten‐eleven translocation
TME: tumor microenvironment
TNBC: triple‐negative breast cancer
Treg: regulatory T cell

## 1 Introduction

Epigenetic mechanisms refer to heritable and reversible changes in gene expression that occur without alterations in the underlying DNA sequence [1]. These include DNA methylation, histone modifications, noncoding RNAs, and epitranscriptomic modifications. These mechanisms coordinate gene expression programs in a temporally and spatially controlled manner, enabling cells to respond to developmental cues and environmental signals [2]. Consequently, epigenetic dysregulation leads to various disease states, including cancer [3]. Epigenetic deregulation drives tumor initiation and progression by altering cellular programs associated with proliferation, survival, differentiation, plasticity, and therapy resistance as well as by regulating communication between cancer cells and the tumor microenvironment (TME) [4, 5]. Cancer cell‐intrinsic epigenetic alterations modify the TME through tumor‐derived signals, while TME‐derived signals reciprocally reshape the epigenetic landscape of cancer cells [6, 7, 8].

In this review, we discuss recent advances in epigenetic regulation of crosstalk between cancer cells and the TME, with a particular focus on mechanisms that coordinate tumor progression and metastasis.

## 2 Epigenetic regulation

Multiple epigenetic mechanisms cooperate to regulate gene expression, including DNA methylation, histone modifications, noncoding RNAs (ncRNAs), and RNA modifications, all of which have been extensively reviewed elsewhere [9, 10, 11, 12]. Therefore, this section briefly discusses selected key mechanisms relevant to this review.

DNA methylation is generally associated with transcriptional repression and mediated by the actions of two classes of enzymes: writers, DNA methyltransferases (DNMTs), and erasers, the ten‐eleven translocation (TET) family [13, 14, 15]. Histone modifications are also mediated by several classes of writers and erasers [16]. These modifications, collectively referred to as the ‘histone code’, can either promote or repress transcription in a context‐dependent manner [16]. For example, acetylation of H3 and H4 as well as methylation of H3K4, H3K20, H3K36, H3K79, H3R17, and H4R3 are histone marks associated with transcriptional activation [17, 18, 19]. These marks are recognized by reader proteins, which recruit transcriptional machinery and establish an open chromatin state [20]. Conversely, repressive histone modifications, including deacetylation of H3 and H4 and methylation of H3K27, contribute to chromatin compaction and transcriptional silencing [21, 22, 23, 24]. Beyond histone methylation and acetylation, additional histone modifications, including ubiquitination, lactylation, and crotonylation, contribute to chromatin dynamics and transcription [25, 26]. For example, H2AK119 ubiquitination promotes chromatin compaction and transcriptional repression, whereas H2BK120 ubiquitination facilitates transcriptional elongation. Moreover, H3K18 lactylation and H3K18/H3K27 crotonylation are associated with an open chromatin state and gene transcription.

Over the past decade, noncoding RNAs, including microRNAs (miRNAs) and long noncoding RNAs (lncRNAs), have emerged as key regulators of epigenetic gene control [27]. miRNAs primarily function in the suppression of target genes by promoting RNA degradation and/or inhibiting translation of target mRNAs [28, 29]. lncRNAs are broadly classified into circular (circRNAs) and linear RNAs. CircRNAs primarily function as miRNA sponges and modulate the activity of RNA‐binding proteins through direct interactions. Linear lncRNAs can also function as competing endogenous RNAs (ceRNAs) [30, 31, 32]. In addition, linear lncRNAs regulate gene expression through multiple epigenetic mechanisms, including guiding chromatin‐modifying complexes to specific genomic loci, modulating chromatin organization, and facilitating enhancer–promoter interactions, thus shaping transcriptional programs [11].

More recently, epigenetic regulation has been expanded to include epitranscriptomic regulation mediated by various RNA modifications, such as N1‐methyladenosine (m1A), N6‐methyladenosine (m6A), and 5‐methylcytosine (m5C) [33], which regulate key biological processes by modulating RNA stability, splicing, degradation, and translation [34]. Among these, m6A is dynamically regulated by writer complexes (e.g., METTL3 and METTL14), erasers (e.g., FTO and ALKBH5), and readers (e.g., YTHDC1 and YTHDF family members) and is the most extensively studied RNA modification in the context of cancer [35].

Collectively, these epigenetic and epitranscriptomic mechanisms orchestrate multiple regulatory layers to control gene expression in a temporally and spatially coordinated manner. Importantly, dysregulation of these processes not only drives aberrant cellular states but also underlies dynamic interactions between cancer cells and the tumor microenvironment, which is discussed in the following sections.

## 3 Tumor microenvironment dynamics in cancer progression

Cancer initiation and progression are driven by continuous interactions between malignant cells and the surrounding stromal components within their local tissue environment. This environment, known as the tumor microenvironment (TME), is a critical regulator of tumor development and progression. The TME is composed of multiple components, including the extracellular matrix (ECM), stromal, and immune cells [36, 37]. Bidirectional crosstalk between cancer cells and the TME through diverse mechanisms collectively supports tumor progression [36, 37]. In this section, we discuss the components of the TME and their roles in cancer progression.

### 3.1 Cancer‐associated fibroblasts (CAFs)

In many solid tumors, CAFs play critical roles in tumorigenesis, cancer progression, and metastasis through multiple mechanisms [38, 39]. Specifically, CAFs produce ECM components as well as a variety of growth factors and cytokines, promoting cancer cell proliferation, migration, angiogenesis, and immunosuppression [40, 41, 42]. Notably, recent studies in pancreatic cancer have revealed significant heterogeneity among CAF populations, including myofibroblastic CAFs (myCAFs), inflammatory CAFs (iCAFs), antigen‐presenting CAFs (apCAFs), and metabolic CAFs (meCAFs), which exhibit distinct spatial distributions and IL‐6 expression profiles. For example, myCAFs display low IL‐6 expression and localize in close proximity to cancer cells, whereas iCAFs exhibit high IL‐6 expression and are located more distally from tumor cells [43, 44, 45]. In addition, meCAFs are characterized by elevated glycolytic activity [43, 44, 45].

### 3.2 Immune cells

A variety of immune cells, including T cells, natural killer cells (NK cells), B cells, macrophages, dendritic cells, and myeloid‐derived suppressor cells (MDSCs), are another major component of the TME [46, 47]. Among these, T cells play a key role in the regulation of tumor immunity, in which CD8^+^ T cells and regulatory T cells (Tregs) exert anti‐ and protumor effects, respectively [48]. Tumors are often characterized by dysfunctional CD8^+^ T cells and the accumulation of immunosuppressive T‐cell populations [49, 50]. In addition, NK cells play a role in tumor immune surveillance [51]. The role of B cells in cancer remains less well‐defined, but recent studies suggest that memory B cells are enriched in immune checkpoint blockade (ICB) responders [52].

Macrophages are another class of immune cells extensively studied for their roles in cancer progression. Two major classes of macrophages include pro‐inflammatory M1‐like and immunosuppressive M2‐like populations [53]. Specifically, M1‐like macrophages exert antitumor effects by producing inflammatory cytokines, enhancing antigen presentation, and supporting T‐cell–mediated immune responses. In contrast, M2‐like tumor‐associated macrophages (TAMs) promote tumor progression by secreting immunosuppressive cytokines, leading to T cell and NK cell dysfunction [54]. Furthermore, immune‐independent functions of M2, such as promoting cancer cell proliferation, invasiveness, and angiogenesis, have been reported [55, 56].

Finally, MDSCs were originally characterized as promoting cancer progression by suppressing T‐cell activation through the production of inhibitory cytokines, the depletion of essential nutrients, and the generation of reactive oxygen species (ROS) [57]. Similar to M2‐like macrophages, however, MDSCs also play a tumor‐promoting role through immune‐independent mechanisms including angiogenesis [58, 59]. Moreover, MDSC activity is modulated not only by cancer cells but also by CAFs, NK cells, and macrophages [60, 61, 62]. Collectively, these TME components function cooperatively to promote cancer progression, highlighting the importance of targeting the TME for effective cancer therapy.

## 4 Epigenetic governance of cancer cell plasticity

Epigenetic mechanisms play a central role in cancer development and progression by orchestrating cancer cell plasticity, including stemness, migration, and drug resistance. In this section, we discuss how epigenetic regulation governs these processes during tumor progression (Fig. 1).

**Fig. 1:**
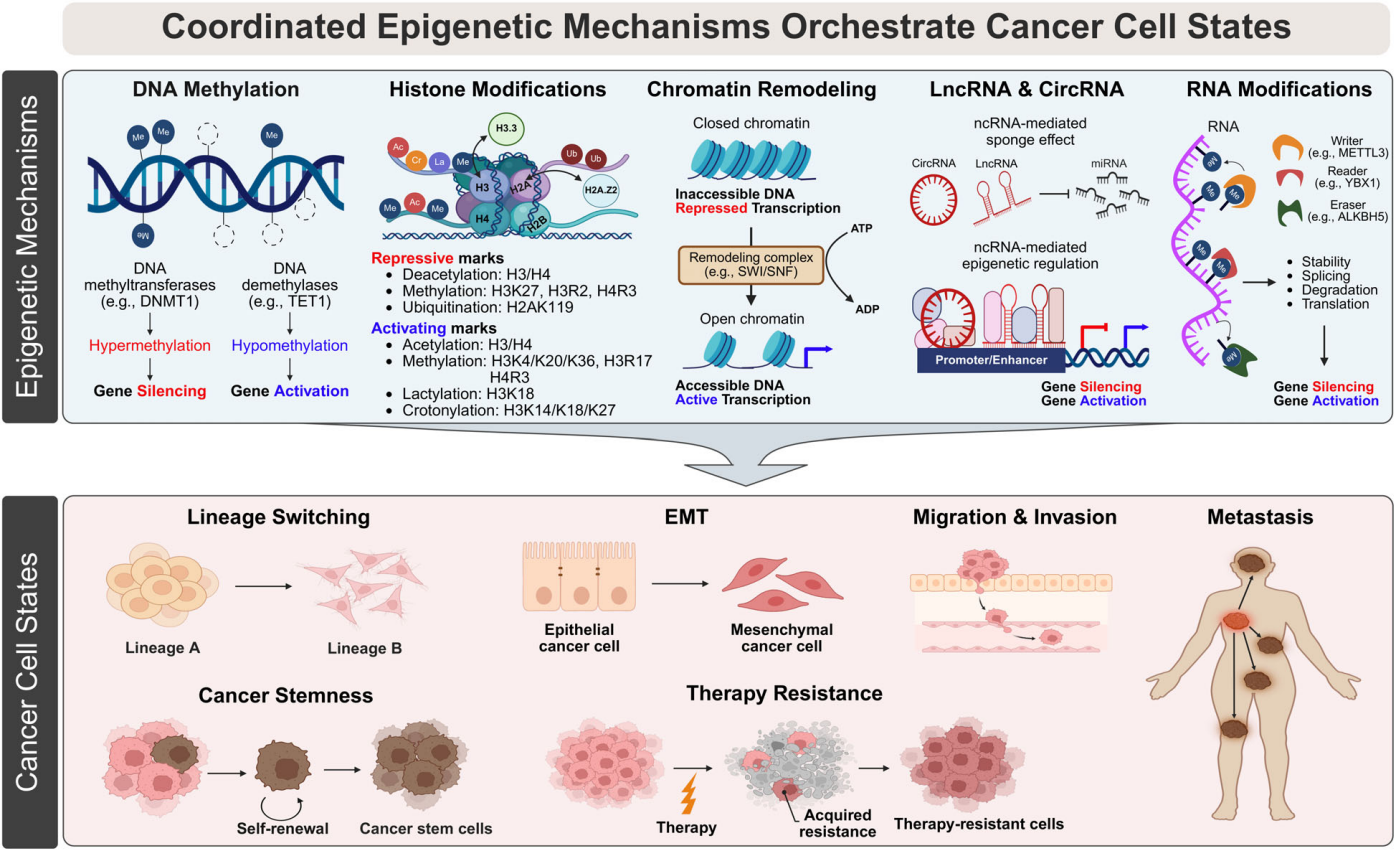
Epigenetic regulation of diverse cancer cell states. Diverse epigenetic mechanisms, including DNA methylation, histone modifications, chromatin remodeling, noncoding RNAs, and RNA modifications, reprogram cancer cell states, thereby driving malignant cellular programs such as lineage switching, stemness, therapy resistance, EMT, migration and invasion, and metastatic progression. Ac, acetylation; ADP, adenosine diphosphate; ATP, adenosine triphosphate; circRNA, circular RNA; Cr, crotonylation; EMT, epithelial–mesenchymal transition; La, lactylation; lncRNA, long noncoding RNA; Me, methylation; miRNA, microRNA; ncRNA, noncoding RNA; Ub, ubiquitination.

### 4.1 Chromatin modification‐mediated regulation of cancer cell plasticity

#### 4.1.1 Stemness and cell state transitions

The acquisition and maintenance of stem‐like traits, lineage‐specific identities, and EMT‐like phenotypes are crucial for cancer progression and are driven by epigenetic reprogramming. For example, DNA methylation silences WNT target genes, such as LGR5 and ASCL2, suppressing stemness‐associated gene signatures in colorectal cancer (CRC) [63], whereas in breast cancer, hypomethylation at enhancer regions bound by lineage‐defining factors, including ERα, FOXA1, and GATA3, helps establish ER^+^‐luminal lineage‐specific transcriptional states by activating estrogen target genes, including progesterone receptor PGR [64]. Histone modifications also provide additional regulation in the promotion of stemness and EMT [65, 66]. For example, KAT7‐mediated H3K14 crotonylation activates fatty acid metabolism genes, including CPT1A, and promotes cancer stem cell self‐renewal [67]. In addition, G9a‐mediated H3K9 methylation and RNF2‐mediated H2AK119 mono‐ubiquitination at the E‐cadherin promoter suppress E‐cadherin transcription in breast cancer (BC) and hepatocarcinoma, respectively, whereas DOT1L‐mediated H3K79 methylation at the SNAIL and ZEB1 promoters upregulates their expression in CRC, promoting EMT [68, 69, 70].

Chromatin architecture further shapes cancer cell states by regulating chromatin accessibility and transcriptional programs. Supporting this, in glioblastoma (GBM), invasive cancer stem cell states exhibit enriched FOX motif accessibility, whereas luminal and basal breast cancer cells display increased chromatin accessibility at the binding motifs for lineage‐associated transcription factors such as ESR1 and SOX members, respectively [71, 72]. Moreover, incorporation of histone variants such as H2A.Z2 at the PLK1 promoter and H3.3 at the ZEB1 and SOX9 promoters alters chromatin accessibility and induces stemness‐ or EMT‐associated transcriptional programs, promoting malignant progression [73, 74].

Collectively, diverse epigenetic mechanisms drive dynamic transitions between different cell states during cancer development and progression by regulating state‐specific chromatin accessibility and transcriptional programs.

#### 4.1.2 Migration and invasion

Migration and invasion are key processes that directly enable cancer cell dissemination, and epigenetic modifications are major regulators of these processes. In several cancers, DNA methylation promotes invasion by silencing anti‐invasive genes. For example, DNA methylation at proteinase inhibitor TIMP2 and TIMP3 promoters silences their transcription, facilitating migration and invasion [75, 76]. In non‐small‐cell lung cancer (NSCLC), DNMT3A‐dependent silencing of FOXA2 enhances invadopodia formation and supports migratory and invasive capacity [77]. Histone modifiers also regulate invasion‐associated transcriptional programs. In breast cancer, UTX cooperates with MLL4 to activate invasive gene expression, including MMP9 and MMP11, by decreasing H3K27 and increasing H3K4 methylation at their promoters, whereas KDM3A removes repressive H3K9 methylation at pro‐invasive genes MMP9 and S100A4 to promote invasion [78, 79]. Emerging histone marks also activate invasion‐related gene programs. In head and neck cancer, the crotonyl‐CoA‐producing enzyme GCDH maintains H3K27 crotonylation at the SPARC promoter, activating invasion‐associated transcriptional programs. In BC, LDHA upregulation by the potassium channel protein KCNK1 promotes H3K18 lactylation by providing lactate, facilitating cancer cell invasion and metastasis [80, 81].

Although distinct epigenetic regulators target different genes in a cancer type‐specific manner, these mechanisms ultimately converge on transcriptional programs that promote intrinsic cell motility and ECM remodeling, leading to cancer cell invasiveness.

#### 4.1.3 Therapy resistance

Chromatin reprogramming is a major mechanism underlying the acquisition of therapy resistance. During therapeutic adaptation, a subset of cancer cells can adopt a reversible drug‐tolerant program marked by distinct chromatin changes [82]. For example, increased repressive histone methylation, particularly H3K9me3 at LINE‐1 loci, suppresses drug‐induced LINE‐1 expression and promotes survival of drug‐tolerant subpopulations [83]. This process is also supported by histone modifiers. KDM5 contributes to therapeutic resistance by preserving transcriptional heterogeneity within cancer cell populations, while chemotherapy‐induced KDM6A activates an OCT4‐associated transcriptional program to enhance BC stemness [84, 85]. Adaptive resistance is further reinforced by altered chromatin architecture. For example, mSWI/SNF complexes alter chromatin accessibility at distal enhancer regions of genes, including MMP13 and BMP4, leading to tyrosine kinase inhibitor resistance in EGFR‐mutant lung cancer [86]. Likewise, prolonged FGFR inhibitor treatment induces loss of BRG1 occupancy at YAP‐associated enhancers, leading to reactivation of YAP target gene expression and consequent resistance to FGFR inhibitors [87]. In addition, loss of ARID1A in ER^+^‐BC disrupts luminal gene regulatory networks, leading to basal‐like transition that diminishes sensitivity to endocrine therapy [88]. Together, these diverse epigenetic mechanisms dynamically reshape cancer cell states and invasive behavior, enabling tumor progression and therapy adaptation.

### 4.2 Epitranscriptomic regulation of cancer progression and metastasis

Accumulating studies have implicated noncoding RNAs and RNA modifications in the regulation of cancer cell plasticity and tumor progression by coordinating various histone modifications and chromatin remodeling. Here, we focus on how linear lncRNAs, circRNAs, and RNA modifications contribute to these processes.

#### 4.2.1 Linear lncRNA

LncRNAs have emerged as epigenetic regulators of cancer cell‐state phenotypes through interactions with chromatin‐modifying machinery at specific genomic loci [89]. For example, LAMP5‐AS1 enhances stemness in MLL leukemia by directly binding to DOT1L and facilitating H3K79 methylation at HOXA9, HOXA10, and MEIS1, while MIR31HG reinforces stem‐like phenotypes in NSCLC by recruiting a WDR5/MLL3/P300 chromatin‐modifying complex at the GLI2 locus [90, 91]. LncRNAs also contribute to EMT by altering chromatin states at EMT‐associated genes. While MAHAC supports hypoxia‐induced EMT by coordinating an H4K5ac‐associated remodeling complex that activates TWIST1 and ZEB1 in NSCLC [92], HOTAIR interacts with PRC2 and induces a global acetylation‐to‐methylation switch on H3K27, suppressing E‐cadherin expression in gastric cancer [93]. Furthermore, lncRNA‐mediated chromatin modifications contribute to migration and invasion. In prostate cancer, HOTTIP directs WDR5 to the HOXA9 promoter, inducing H3K4me3 and its expression, whereas in gastric cancer, GClnc1 functions as a scaffold for WDR5 and KAT2A to increase H3K4me3 and H3K9ac at the SOD2 promoter, upregulating its expression; both mechanisms promote migration, invasion, and metastasis [94, 95]. Moreover, lncRNA‐mediated chromatin modification contributes to lineage plasticity and therapy‐induced cell‐state transitions. In neuroblastoma, a small nucleolar RNA SNHG1 interacts with HDAC1/2 to repress differentiation‐associated genes, including NR2F1, NEFH, and YAP, maintaining a proliferative cell state [96]. In prostate cancer, lncRNA‐p21 activates EZH2/STAT3 signaling during enzalutamide‐induced neuroendocrine differentiation [97].

Collectively, linear lncRNAs play a key role as scaffolds for chromatin‐modifying complexes that mediate epigenetic reprogramming of cancer cells, regulating their stemness, EMT, invasion, and therapy resistance.

#### 4.2.2 Circular RNA

Although less extensively studied, circRNAs have emerged as important epigenetic regulators of cancer cell stemness, invasiveness, and therapeutic resistance by directing chromatin modifiers to specific target genes, similar to the mechanisms employed by lncRNAs. For example, CircCTIC1 interacts with BPTF and recruits the NURF complex to the MYC promoter, enhancing its expression and tumor‐initiating capacity in colon cancer [98]. Similarly, cia‐MAF increases MAFF expression to sustain self‐renewal by recruiting the KAT5 complex and promoting local histone acetylation at the MAFF promoter in liver cancer [99]. Moreover, circRNA‐mediated epigenetic reprogramming promotes cancer cell migration and invasion. In BC, FECR1 facilitates invasion by increasing FLI1 expression through TET1 recruitment and DNMT1 downregulation, while in melanoma, circROR1 promotes invasion by upregulating CCNE1 via recruitment of KAT2A to promote H3K9 acetylation [100, 101]. CircRNAs also promote therapy resistance through epigenetic programs supporting survival under therapeutic pressure. For example, hsa_circ_0007919 binds FOXA1 and TET1 to activate LIG1 transcription, enhancing DNA damage response and gemcitabine resistance in pancreatic ductal adenocarcinoma (PDAC) [102].

#### 4.2.3 Epitranscriptomic modifications of RNA

Epitranscriptomic modifications, particularly m6A and 5‐methylcytosine (m5C), are increasingly recognized as key regulators of cancer cell stemness, invasiveness, and therapeutic resistance. For example, reduced m6A levels are observed in GBM stem cells and contribute to the upregulation of several oncogenic transcripts, such as ADAM19 and KLF4, increasing self‐renewal and tumor initiation [103]. In BC, hypoxia‐induced activation of ALKBH5 expression stabilizes NANOG by demethylating the 3′‐UTR of NANOG mRNA, leading to increased stemness [104]. In addition, METTL3‐induced m6A methylation of EGR1 mRNA enhances its stability and subsequently activates SNAIL expression, promoting invasion and metastasis in esophageal squamous cell carcinoma (ESCC) [105]. In gastric cancer, increased m6A modification of ZMYM1 mRNA stabilizes the transcript, enabling ZMYM1 to repress E‐cadherin and thereby facilitate EMT and invasion [106].

m5C also contributes to malignant phenotypes. In head and neck squamous cell carcinoma, the m5C writer NSUN3 induces m5C modification of mitochondrial tRNA^MET^, promoting mitochondrial protein translation and sustaining oxidative phosphorylation, facilitating invasion and metastasis [107]. Moreover, YBX1, an m5C reader protein, is overexpressed in ESCC and binds m5C‐modified SMOX mRNA, increasing its stability and leading to enhanced stemness and metastasis [108]. In NSCLC, NSUN2‐mediated m5C hypermethylation of QSOX1 and NRF2 mRNAs enhances their stability and protein expression, promoting therapeutic resistance [109, 110].

Collectively, altered RNA modifications in cancer cells control post‐transcriptional gene expression by regulating RNA stability and translation, contributing to cancer cell plasticity, metastatic progression, and therapeutic resistance.

## 5 Epigenetic reprogramming of the tumor microenvironment

In addition to cancer cell‐intrinsic epigenetic alterations, epigenetic remodeling of stromal and immune components within the TME promotes cancer progression by facilitating immune evasion, cancer cell proliferation, and invasion. These changes are largely driven by tumor‐ and microenvironment‐derived cues, establishing a tumor‐supportive TME [47, 111, 112]. The following section highlights the key epigenetic mechanisms underlying TME reprogramming during cancer progression and metastasis (Fig. 2).

**Fig. 2:**
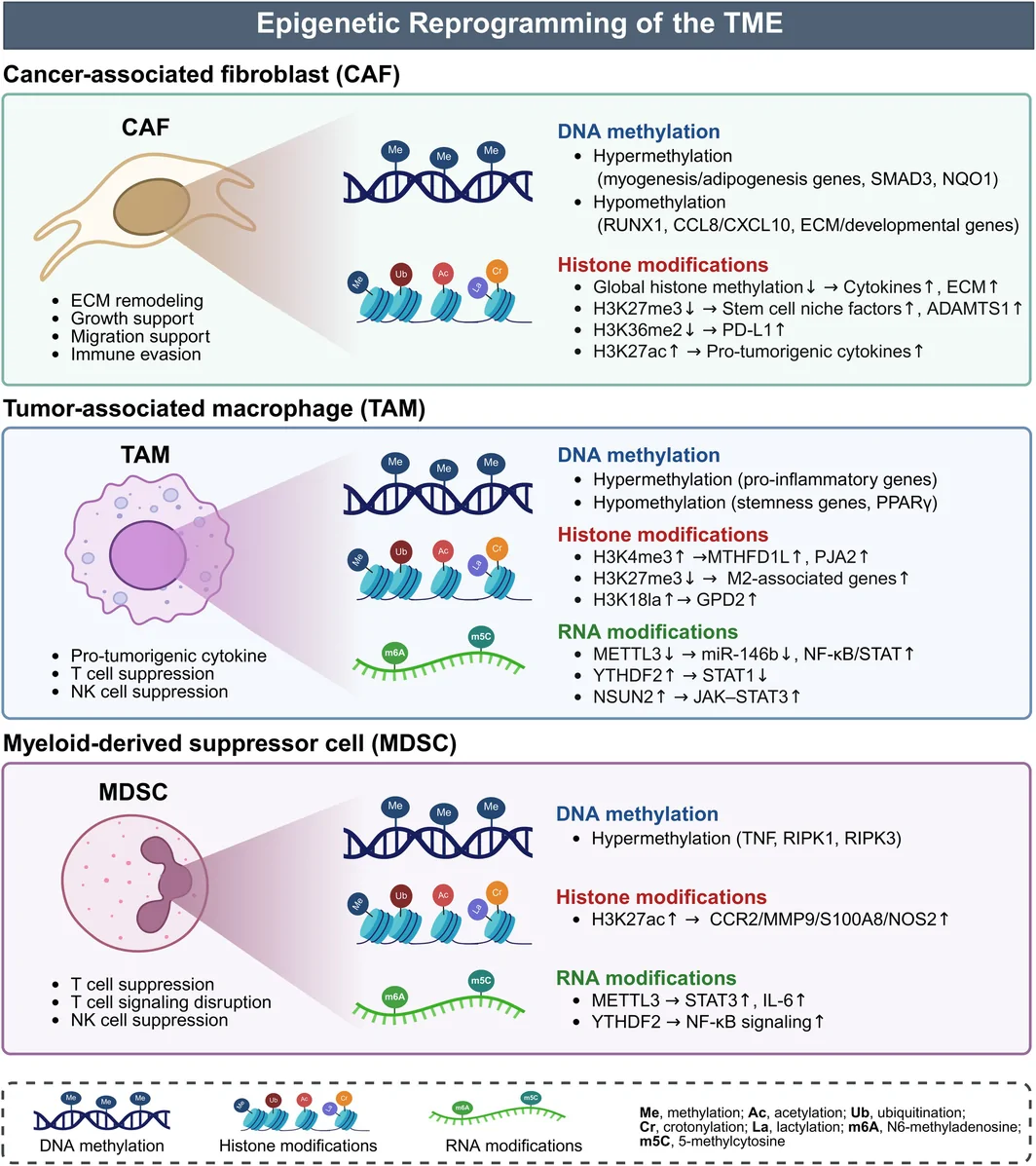
Epigenetic reprogramming of the tumor microenvironment. The figure summarizes epigenetic regulation of major TME components, including cancer‐associated fibroblasts (CAFs), tumor‐associated macrophages (TAMs), and myeloid‐derived suppressor cells (MDSCs). In each cell type, distinct epigenetic alterations establish characteristic transcriptional and functional states associated with stromal support and immune suppression, collectively establishing a tumor‐supportive microenvironment. Representative examples of these epigenetic alterations and their associated consequences are shown. Ac, acetylation; CAF, cancer‐associated fibroblast; Cr, crotonylation; ECM, extracellular matrix; La, lactylation; m6A, N6‐methyladenosine; m5C, 5‐methylcytosine; MDSC, myeloid‐derived suppressor cell; Me, methylation; NK, natural killer cell; TAM, tumor‐associated macrophage; TME, tumor microenvironment; Ub, ubiquitination.

### 5.1 Epigenetic regulation of cancer‐associated fibroblasts (CAFs)

Fibroblasts represent a major noncancer component of the TME and exhibit epigenetic reprogramming during their transition into cancer‐associated fibroblasts (CAFs), sustaining their tumor‐promoting state [113].

#### 5.1.1 DNA methylation‐mediated regulation of CAF function

Genome‐wide analyses indicate that altered DNA methylation is a prominent feature of CAFs, primarily occurring at regulatory regions of genes involved in developmental programs, metabolism, and inflammatory responses, promoting establishment of a tumor‐promoting environment [114, 115, 116, 117]. The DNA methylation landscape of CAFs varies across tumor types and correlates with disease progression [116, 118, 119]. In prostate cancer, altered methylation in CAFs affects genes involved in developmental programs, including GATA and FOX transcription factor networks, as well as pathways related to metabolism, ECM organization, and cell adhesion, promoting cancer progression [116, 118]. In breast CAFs, RUNX1 is hypomethylated and upregulated. Coculture with BCCs induces RUNX1 and a RUNX1‐dependent stromal program during fibroblast‐to‐CAF conversion, and activation of this signature is associated with poor clinical outcome [120]. DNA methylation of metabolic genes in CAFs also contributes to organ‐specific metastasis. For example, CAFs from PDAC liver metastases exhibit hypermethylation at NQO1 and ALDH1A3, accompanied by broad metabolic gene suppression and a relatively homogeneous iCAF‐like phenotype, which has been proposed to contribute to the aggressive behavior of liver metastases [121]. Furthermore, hypermethylation‐mediated suppression of SMAD3 in lung CAFs attenuates TGF‐β1‐driven fibrotic programs, reducing the stromal and tumor response to nintedanib [122].

#### 5.1.2 Histone modification‐mediated regulation of CAF function

Besides DNA methylation, altered histone modifications in CAFs, particularly H3 methylation and acetylation marks, have been implicated in cancer progression [123, 124, 125]. For example, in gastric cancer CAFs, reduced H3K27me3 is enriched at genes associated with stem cell niche regulation, tissue development, and stromal–epithelial interactions, leading to a tumor‐promoting CAF phenotype [125]. Similarly, loss of H3K27me3 in BC CAFs promotes ADAMTS1 secretion, thus promoting tumor invasion [123]. Other histone methylation marks also contribute to CAF function. In BC, H3K36 demethylase KDM2A is upregulated in CAFs, where it promotes p53‐mediated senescence, senescence‐associated secretory phenotype (SASP) cytokine production, and PD‐L1 expression, enhancing tumor cell proliferation [126]. In addition, increased expression of nicotinamide N‐methyltransferase (NNMT) in CAFs from high‐grade serous ovarian carcinoma metastases has been reported. Elevated NNMT causes cellular SAM depletion, resulting in reduced global levels of H3K4me3 and H3K27me3, leading to transcriptional reprogramming of CAFs and promoting tumor progression, migration, and metastasis [124]. Furthermore, elevated H3K27ac levels at genes involved in the production of protumorigenic cytokines have been observed in CAFs from multiple cancer types, including gastric, pancreatic, breast, colon, and lung cancers [127, 128, 129, 130, 131].

Together, DNA methylation and histone modifications function cooperatively to establish and maintain CAF phenotypes while enabling CAF adaptation to tissue‐specific microenvironments through the regulation of developmental, inflammatory, and metabolic transcriptional programs, establishing tumor‐promoting TME.

### 5.2 Epigenetic reprogramming of TAM polarization

Macrophages represent a major immune component of the TME and undergo extensive functional and epigenetic reprogramming during TAM polarization [132, 133].

#### 5.2.1 DNA methylation‐mediated regulation of TAM polarization

Aberrant DNA methylation in TAMs contributes to immune evasion by promoting TAM polarization [132, 134]. For example, DNMT overexpression in TAMs causes epigenetic silencing of pro‐inflammatory and immune‐stimulatory genes, reinforcing immunosuppressive phenotypes [135]. In addition, tumor‐derived redHMGB1 induces the formation of stem cell–like memory macrophages (SMMs) by promoting the recruitment of TET2 to stemness‐associated genes. Subsequently, this TET2‐dependent remodeling establishes a stem cell–like memory macrophage state with enhanced protumorigenic activity [136]. Adding another layer of complexity, thymine DNA glycosylase (TDG)‐mediated DNA demethylation has been shown to promote M2 polarization. Specifically, tumor‐derived arginine enhances polyamine production, which then induces TDG‐dependent demethylation of PPARγ, driving M2 polarization [137].

#### 5.2.2 Histone modification‐mediated regulation of TAM polarization

Histone modifications also regulate TAM polarization by modulating diverse inflammatory transcriptional programs. For example, SMYD3 and KDM5A, a writer and an eraser of H3K4 methylation, respectively, have been shown to regulate TAM polarization. Specifically, SMYD3 enhances folate metabolism through H3K4 methylation at the MTHFD1L locus, which in turn suppresses the expression of M1‐associated genes and promotes an immunosuppressive TAM phenotype [138]. Similarly, in gastric cancer, KDM5A suppresses the expression of the E3 ubiquitin‐protein ligase PJA2 through H3K4 demethylation‐dependent transcriptional repression, stabilizing KSR1, a scaffold protein that facilitates MAPK signaling, ultimately leading to M2 polarization and cancer progression [139]. H3K27 methylation dynamics also play a key role in macrophage polarization. KDM6A supports M1 polarization by promoting H3K27me3 removal and the expression of pro‐inflammatory cytokine genes under altered ROS conditions, whereas IL‐4–induced KDM6B induces M2‐associated gene expression through H3K27me3 demethylation [140, 141]. Furthermore, lactate from cervical cancer cells increases H3K18 lactylation in macrophages, leading to GPD2 upregulation, a key regulator of glucose oxidation and inflammatory responses, promoting M2‐like macrophage polarization [142].

#### 5.2.3 Epitranscriptomic regulation of TAM polarization

Emerging studies have demonstrated context‐dependent roles of m6A and m5C modifiers in macrophage polarization in multiple cancer types [134, 143, 144, 145]. METTL3 exerts tumor‐suppressive functions through regulation of miRNA maturation and pro‐inflammatory cytokine production [146, 147]. Specifically, deletion of METTL3 in CRC inhibits the maturation of miR‐146b, a key regulator of the pro‐inflammatory state, promoting M2 polarization and tumor progression [147]. In addition, deletion of METTL3 in myeloid cells activates the nuclear factor kappa B (NF‐κB) and STAT signaling pathways. This increases TAMs, ultimately promoting melanoma and lung cancer progression and metastasis [146]. m6A readers, particularly YTHDF family members, also regulate TAM function. For example, YTHDF2 promotes the degradation of m6A‐modified transcripts such as STAT1, suppressing pro‐inflammatory gene expression and reinforcing M2‐like polarization, whereas its inhibition reprograms TAMs toward a more pro‐inflammatory, antitumor phenotype [148]. In addition, NSUN2‐mediated m5C methylation of SOCS3 transcripts reduces their stability and impairs their export from the nucleus, leading to activation of JAK/STAT3 signaling and ultimately driving M2 polarization [149].

### 5.3 Epigenetic reprogramming of MDSCs

Epigenetic mechanisms, especially DNA methylation and histone acetylation, have also been reported to regulate MDSC migration, differentiation, and immunosuppressive function [150, 151]. Supporting this, a recent study demonstrated that combined inhibition of DNMTs and HDACs reduces the expression of CCR2 and CXCR2 in MDSCs, which attenuates their infiltration in the lung and disrupts premetastatic niche formation [152]. RNA modifications also control MDSC function. For example, METTL3 is upregulated in MDSCs and promotes activation of the STAT3 pathway, leading to increased IL‐6 production and suppression of T‐cell activation, promoting colorectal cancer progression [153]. In addition, therapy‐induced upregulation of YTHDF2 in MDSCs enhances degradation of m6A‐modified transcripts encoding negative regulators of NF‐κB signaling, resulting in activation of the NF‐κB pathway and promoting MDSC infiltration and differentiation [154].

In summary, epigenetic reprogramming across multiple stromal and immune components of the TME establishes a tumor‐supportive microenvironment. Although distinct epigenetic mechanisms promote CAF activation, TAM polarization, and MDSC function, these processes ultimately converge on key biological outcomes, including stromal activation, immune evasion, and chronic inflammation, driving tumor progression.

## 6 Epigenetic coordination of cancer cell–tumor microenvironment crosstalk

Epigenetic alterations in cancer cells not only regulate cancer cell‐intrinsic phenotypes but also reshape cancer cell–TME crosstalk by controlling the production and composition of tumor‐derived signals, including exosomal noncoding RNAs, cytokines, and metabolites. These signals subsequently induce diverse epigenetic, transcriptional, and metabolic rewiring in TME components, fostering a tumor‐supportive TME (Fig. 3).

**Fig. 3:**
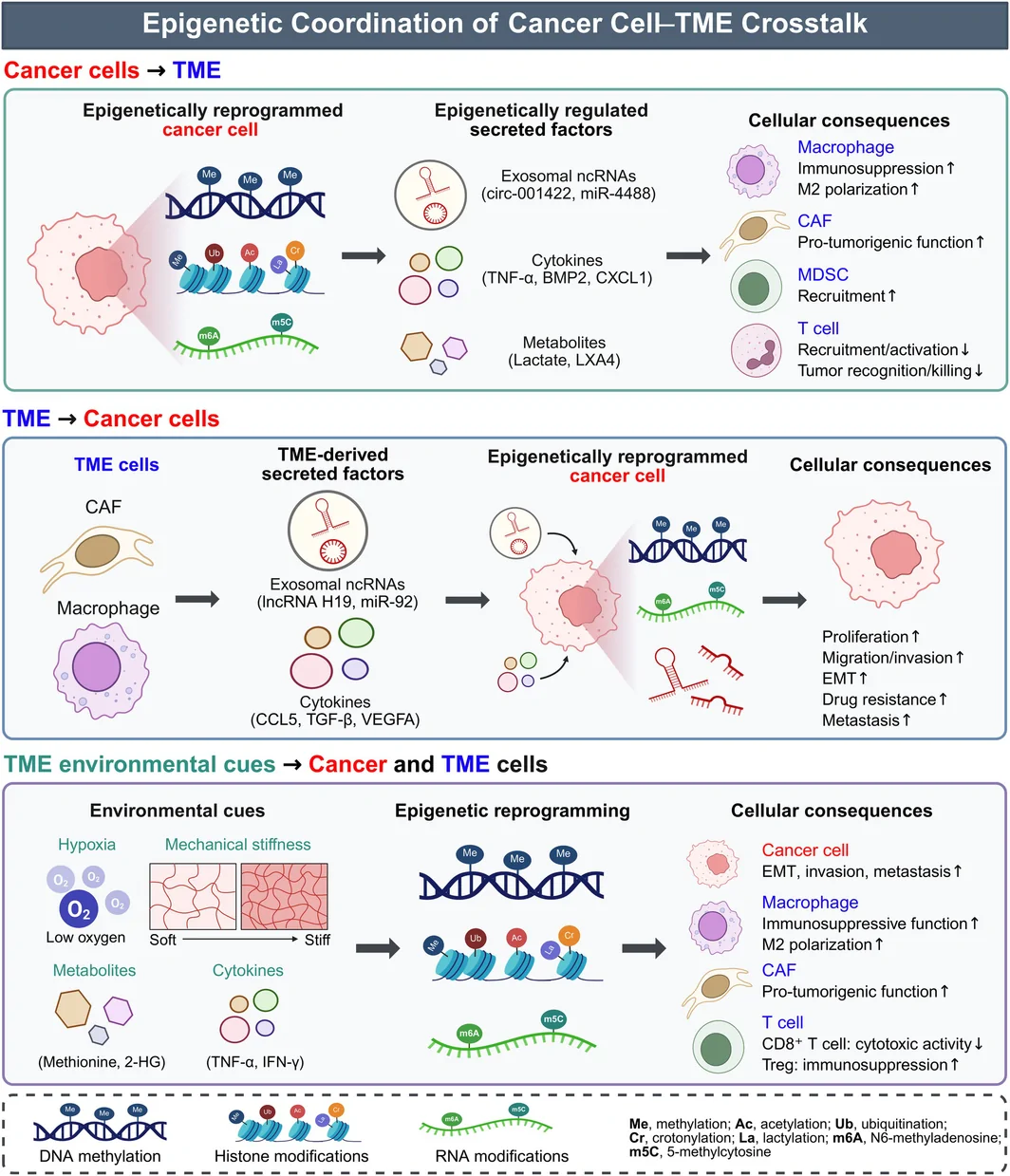
Epigenetic coordination of cancer cell–TME crosstalk. The figure illustrates bidirectional epigenetic communication between cancer cells and the TME. Cancer cell‐intrinsic epigenetic changes shape the TME through exosomal noncoding RNAs, cytokines, and metabolites (top panel) whereas TME‐derived signals reciprocally reprogram cancer cells to promote EMT, proliferation, migration and invasion, drug resistance, and metastasis (middle panel). Environmental cues such as hypoxia, metabolite competition, oncometabolites, cytokines, and matrix stiffness further modulate epigenetic changes in cancer cells and TME components (bottom panel). 2‐HG, 2‐hydroxyglutarate; Ac, acetylation; CAF, cancer‐associated fibroblast; Cr, crotonylation; EMT, epithelial–mesenchymal transition; La, lactylation; lncRNA, long noncoding RNA; m6A, N6‐methyladenosine; m5C, 5‐methylcytosine; MDSC, myeloid‐derived suppressor cell; Me, methylation; miRNA, microRNA; ncRNA, noncoding RNA; TME, tumor microenvironment; Treg, regulatory T cell; Ub, ubiquitination.

### 6.1 Cancer cell epigenetic programs shaping the TME

#### 6.1.1 Exosomal noncoding RNAs (ncRNAs)

Accumulating evidence demonstrates that tumor‐derived exosomal ncRNAs play an important role in modulating TME, particularly in reprogramming TAMs. Exosomal ncRNAs are internalized by TAMs and regulate epigenetic enzymes within TAMs [155, 156, 157]. For example, exosomal circ‐001422 in glioma and FGD5‐AS1 in pancreatic cancer physically interact with p300, inducing STAT3 acetylation and subsequent activation of STAT3/NF‐κB signaling, leading to immunosuppressive TAM polarization [155, 156]. In addition, tumor‐derived circRNAs function as miRNA sponges to promote immunosuppressive macrophage phenotypes. For example, exosomal circATP8A1 sequesters miR‐1‐3p and activates the STAT6 axis, inducing M2 polarization and promoting gastric cancer progression [158]. Exosomal miRNAs and lncRNAs also regulate epigenetic enzymes in TAM. BC‐derived exosomal miR‐138‐5p downregulates KDM6B and increases H3K27me3 at pro‐inflammatory gene promoters, promoting M2 polarization [157], while lncRNAs XIST in CRC and HCG18 in gastric cancer promote M2 polarization through regulation of PDGFRα and KLF4, respectively [159, 160].

Furthermore, exosomal ncRNAs also play a role in the metabolic reprogramming of TAM by directly regulating genes involved in glycolysis, lipid metabolism, and fatty acid oxidation in multiple cancer types [161, 162, 163, 164, 165]. For example, in pancreatic cancer, tumor‐derived exosomal miR‐4488 directly targets and suppresses the ER membrane protein RTN3 in macrophages, and subsequently leads to downregulation of FABP5, an important regulator of lipid metabolism, increasing fatty acid oxidation. Similarly, miR‐125b‐5p in melanoma decreases lipid catabolic processes by targeting lysosomal acid lipase A (LIPA), reprogramming TAM metabolism toward a tumor‐promoting phenotype [161, 162]. Collectively, tumor‐derived exosomal ncRNAs function as intercellular messengers that alter the epigenetic and metabolic states of macrophages, promoting immunosuppressive TAM polarization.

#### 6.1.2 Tumor‐derived cytokines and metabolites

Tumor‐derived cytokines and metabolites also modulate epigenetic programs in the TME. For example, TNF‐α expression in ovarian cancer cells (OCCs) is induced by DNA hypomethylation, which subsequently activates the NF‐κB pathway and leads to increased TGF‐α expression in CAFs. This then activates EGFR signaling in OCCs, promoting metastatic colonization [166]. Furthermore, SETD2 deficiency in pancreatic cancer cells upregulates BMP2 expression through increased H3K27 acetylation, driving CAF differentiation toward a lipid‐rich phenotype, which subsequently supplies lipids to cancer cells, promoting tumor progression [167].

Conversely, tumor‐derived cytokines produced by epigenetic changes in cancer cells also govern the recruitment and activation of immune cells within the TME. For example, elevated expression of the acetyl‐lysine reader CECR2 in metastatic BC promotes the expression of a panel of M2‐polarizing cytokines, while p300 mutations in B‐cell lymphoma activate the NOTCH pathway, both of which induce M2 polarization [168, 169]. Furthermore, the RNA demethylase ALKBH5 increases IL‐8 expression in GBM cells, leading to TAM recruitment and polarization [170]. Tumor‐derived cytokines also contribute to MDSC recruitment. One such mechanism involves altered m6A modifiers in various cancers [171, 172, 173, 174]. METTL3 overexpressed in CRC and NSCLC induces cytokine expression, promoting MDSC recruitment, while the m6A reader YTHDF1 activates the NF‐κB pathway by enhancing p65 translation, which induces expression of CXCL1 and promotes MDSC infiltration into the TME [173].

Epigenetic alterations, including diverse histone modifications, in cancer cells also suppress antitumor T‐cell immunity by regulating T‐cell recruitment, activation, and antigen presentation through altered cytokine expression. For example, the H3K9 methyltransferase SETDB1 is overexpressed in melanoma and lung cancer, where it suppresses IFN genes, attenuating T‐cell infiltration and activation [175, 176, 177]. In ovarian cancer, epigenetic alterations mediated by EZH2 and DNMTs suppress Th1‐type chemokines such as CXCL9 and CXCL10, preventing immune cell infiltration [178]. In addition to methylation, lysine acetylation and crotonylation also regulate T‐cell infiltration. HDAC8 is overexpressed in hepatocarcinoma cells and suppresses CCL4 expression [179], while reprogrammed lysine catabolism in GBM stem cells increases intracellular crotonyl‐CoA levels, leading to histone H4K crotonylation, which suppresses type I IFN signaling, both of which reduce CD8^+^ T cell infiltration, promoting tumor immune evasion [180]. Furthermore, cancer cell‐intrinsic histone ubiquitination regulates antitumor immunity by modulating antigen presentation. In melanoma, PCGF1‐mediated H2AK119 mono‐ubiquitination at the MHC‐I promoters silences its transcription, reducing tumor recognition by T cells [181].

Beyond cytokines, tumor‐derived metabolites also contribute to epigenetic reprogramming of TME components, establishing a tumor‐promoting TME. For example, tumor‐derived lactate facilitates CAF formation by inducing α‐ketoglutarate‐dependent TET activation in PDAC [182]. Lactate‐mediated H3K18 lactylation in macrophages in CRC suppresses RARγ transcription, activating the NF‐κB pathway and IL‐6 secretion, whereas H3K18 lactylation in macrophages in hepatocarcinoma increases NUPR1, promoting an immunosuppressive TAM phenotype and cancer progression [183, 184].

These metabolite‐driven effects also extend to epitranscriptomic regulation. In CRC, cancer cell‐derived lactate induces H3K18 lactylation and subsequent upregulation of METTL14 in macrophages, increasing m6A modification of VSIG4 and its expression, which promotes fatty acid oxidation‐dependent M2 polarization of TAMs [185]. Similarly, cancer cell‐derived lipoxin A4 (LXA4) suppresses the m6A writer METTL3 in TAMs, leading to STAT6 activation and M2 polarization, supporting prostate cancer progression [186].

Collectively, epigenetic alterations in cancer cells extend beyond autonomous regulation of cancer cell phenotypes to reshape the TME. By controlling the production of exosomal noncoding RNAs, cytokines, metabolites, and immune‐regulatory molecules, epigenetic changes in cancer cells induce epigenetic, transcriptional, and metabolic reprogramming across stromal and immune cells, reinforcing a tumor‐promoting microenvironment.

### 6.2 TME‐driven epigenetic reprogramming of cancer cells

While the mechanisms by which the TME regulates the epigenetic landscape of cancer cells remain incompletely understood, accumulating evidence supports a role for TME‐derived signals in this process. For example, TME‐derived exosomal ncRNAs contribute to epigenetic reprogramming in cancer cells. In CRC, CAF‐derived exosomal lncRNA H19 acts as a competing endogenous RNA (ceRNA) to sequester miR‐141, activating the β‐catenin pathway and promoting cancer stemness [187]. In BC, CAF‐derived exosomal miRNAs, including miR‐92 and miR‐500a‐5p, promote cancer cell proliferation, invasion, and metastasis by regulating G3BP2 and USP28, respectively [188, 189]. CAF‐derived exosomal miRNAs also contribute to drug resistance in multiple cancers. In ovarian cancer, CAF‐secreted miR‐21 and miR‐98‐5p drive chemoresistance by suppressing apoptosis‐related genes APAF1 and CDKN1A, respectively [190, 191].

TAM‐derived cytokines also contribute to epigenetic alterations in cancer cells. TAM‐derived CCL5 increases DNMT1 expression in gastric cancer cells and promotes tumor growth by suppressing GSN (gelsolin) [192]. CAF‐derived TGF‐β1 induces HOTAIR in breast cancer cells, which promotes H3K27 methylation at the CDK5 inhibitor CDK5RAP1 locus, resulting in increased CDK5 expression and EMT, ultimately enhancing tumor growth and lung metastasis [193]. Furthermore, recent reports suggest that TME‐derived signals also induce epitranscriptomic changes in cancer cells [194, 195]. In NSCLC, CAF‐derived VEGFA increases METTL3 expression and m6A modification of RAC3 mRNA, enhancing its stability and translation to activate the AKT/NF‐κB pathway, promoting tumor cell growth and invasion [196].

Taken together, TME‐derived cytokines and exosomal noncoding RNAs reshape the epigenetic landscape of cancer cells, promoting tumor progression and metastasis.

### 6.3 Environmental control of epigenetic plasticity in cancer and TME

Epigenetic alterations within TME components reciprocally influence cancer cell behavior and immune responses, promoting tumor progression. One of the key drivers of such alterations is hypoxia, which induces epigenetic remodeling across multiple cell types in the TME. For example, hypoxia alters histone methylation at IGF2 and HGF in PDAC CAFs, promoting EMT and tumor progression [197]. Hypoxia suppresses JMJD3 expression in T cells and increases H3K27me3 at the promoters of IL2 and BCL6, which are involved in T‐cell longevity and stemness, respectively, thereby impairing T‐cell function [198]. In BC, hypoxia suppresses the expression of cytotoxic cytokines, including IFN‐γ and TNF‐α, in T cells through coordinated epigenetic regulation involving HIF1α, PRC2, and HDAC1, driving an exhaustion‐like phenotype [199].

Metabolic constraints further contribute to epigenetic regulation of immune cells. For example, competition for key metabolites such as methionine, a donor for S‐adenosylmethionine (SAM), affects histone methylation in T cells; accordingly, overexpression of methionine transporter SLC43A2 in melanoma and colon cancer cells limits methionine availability in T cells, leading to H3K79me2 loss at the STAT5 gene, impairing T‐cell function [200]. Gain‐of‐function mutations in IDH1 in glioma cells increase 2‐hydroxyglutarate (2‐HG), a competitive inhibitor of histone and DNA demethylases, which can be taken up by T cells and suppress their cytotoxic activity [201].

Beyond metabolite competition, TME‐derived metabolites and cytokines modulate the epigenetic landscape of cancer cells and other TME components. For example, CAF‐secreted lactate induces histone lactylation in cancer cells, promoting invasion, metastasis, and chemoresistance [202, 203, 204, 205]. In TNBC, TME‐derived cytokines such as IFN‐γ, TGF‐β, and TNF‐α induce transcriptional programs governed by FOSL2, STAT1, and RUNX3 in TAMs, leading to hypomethylation of CD274 that promotes immunosuppressive phenotypes [132]. TME‐derived metabolites and cytokines also regulate immune cell function through RNA modifications. In melanoma, TME‐derived TNF‐α activates NF‐κB in Tregs, inducing YTHDF2 expression. Upregulated YTHDF2 reinforces NF‐κB signaling by accelerating the decay of m6A‐modified mRNAs encoding negative regulators of NF‐κB, enhancing Treg survival and immunosuppressive function [206].

Finally, mechanical properties of the TME, including ECM stiffness and mechanical stress, contribute to epigenetic reprogramming in cancer cells. In several cancer types, YAP1 functions as a key mechanotransducer and is associated with cancer progression [207]. In gastric cancer, increased ECM deposition correlates with poor prognosis [208] and promotes TET2‐mediated hypomethylation of the YAP1 promoter and upregulation of YAP1 [209]. In PDAC, high matrix stiffness promotes METTL14‐mediated m6A modification of YAP1 mRNA, enhancing stemness and gemcitabine resistance [210]. Mechanical stress within the stiff tumor matrix also modulates the epigenetic states of immune cells, thus promoting immunosuppression. In CD8^+^ T cells, ECM‐induced mechanical stress suppresses cytotoxic gene expression, including IFN‐γ and PRF1, through HDAC3‐mediated reduction of H3K27ac, leading to CD8^+^ T‐cell exhaustion [211]. In TAMs, matrix stiffness accelerates M2‐associated gene expression by promoting STAT6 nuclear translocation through enhanced nuclear pore permeability and increasing chromatin accessibility via inhibition of H3K9 methylation, ultimately promoting M2 polarization within the TME [212].

Collectively, diverse environmental cues within the TME, including hypoxia, metabolic competition, cytokines, metabolites, and mechanical cues, regulate diverse epigenetic mechanisms that reshape gene expression programs across cancer, stromal, and immune cells, ultimately resulting in common biological outcomes that promote cancer progression and therapeutic responses.

## 7 Recent advances in anticancer epigenetic therapeutics

Given the central role of epigenetic regulation in cancer progression and metastasis, epigenetic drugs have emerged as promising anticancer therapies. For example, inhibitors of DNMTs, HDACs, EZH2, and mutant IDH are now used clinically, and their efficacy has been evident in hematologic malignancies, whereas activity in solid tumors remains limited [213]. The modest efficacy of epigenetic therapies in solid tumors is attributed to the epigenetic plasticity of cancer cells and the unique features of the solid cancer TME. The solid tumor TME is characterized by dense ECM deposition, abnormal tumor vasculature, elevated interstitial pressure, and hypoxic conditions, which collectively restrict drug delivery [214]. Furthermore, heterogeneous populations of cancer, stromal, and immune cells that comprise the TME collectively exhibit distinct epigenetic states [215]. Consequently, broad‐acting epigenetic therapies may elicit diverse, sometimes opposing, effects across different cellular components within the TME, limiting therapeutic efficacy. To overcome these challenges, clinical development has shifted from broad epigenetic monotherapy toward more mechanism‐based therapeutic strategies [216].

One major strategy is dual epigenetic targeting. Combinations of DNMT and HDAC inhibitors have been shown to reactivate silenced tumor suppressor genes and modulate the TME [152, 217]. Such strategies have been evaluated in metastatic CRC (NCT01105377) and recurrent advanced NSCLC (NCT00387465). A related approach is the development of single‐molecule dual epigenetic inhibitors, such as JBI‐802, an LSD1/HDAC6 inhibitor in phase I/II clinical evaluation for advanced solid tumors (NCT05268666). Another important strategy is combining epigenetic therapy with immunotherapy, based on the rationale that epigenetic alterations in cancer, immune, and stromal cells cooperatively reinforce the immunosuppressive TME. In this context, DNMT inhibition can induce viral mimicry and enhance tumor immunogenicity [218], while DNA hypomethylating agents may promote antitumor CD8^+^ T‐cell function [219]. Combinations of epigenetic drugs with immune checkpoint inhibitors are being evaluated in solid tumors, including azacitidine plus pembrolizumab (NCT02546986), vorinostat plus pembrolizumab (NCT02638090), and tazemetostat plus durvalumab (NCT04705818). Furthermore, oncometabolite‐targeted strategies highlight the translational potential of mechanism‐based epigenetic therapy in solid tumors. Because mutant IDH produces the oncometabolite 2‐HG and promotes global DNA hypermethylation, dual inhibition of mutant IDH and DNMT has a mechanistic rationale [220]. Although clinical proof of this concept has been established in hematologic malignancies, as evidenced by FDA approval of ivosidenib plus azacitidine for newly diagnosed IDH1‐mutant acute myeloid leukemia, recent approvals of IDH‐targeted therapies in cholangiocarcinoma (NCT02989857) and IDH‐mutant glioma (NCT04164901) further support the therapeutic relevance of this pathway in solid tumors.

## 8 Conclusions and future directions

Epigenetic regulation is a central mechanism governing cancer development and progression through coordinated control of cancer cell‐intrinsic programs and the tumor microenvironment (TME). Multiple layers of epigenetic regulation—including DNA methylation, histone modifications, noncoding RNAs, and epitranscriptomic mechanisms—coordinate cellular plasticity, immune responses, and stromal dynamics to establish protumorigenic states. These regulatory processes involve bidirectional crosstalk between cancer cells and the TME, underscoring the complexity and plasticity of epigenetic states in cancer and the surrounding stroma.

Given the essential role of epigenetic mechanisms on cancer, targeting epigenetic alterations has emerged as a promising therapeutic strategy, with a number of clinical trials demonstrating significant advances. However, achieving cell type‐specific targeting and minimizing systemic toxicity remain key challenges. To overcome these limitations, future research warrants integrated approaches encompassing single‐cell epigenomics, temporal dynamics, spatial heterogeneity, and reversible epigenetic reprogramming in the TME, combined with functional studies. For example, although multiple CAF subtypes (e.g., myCAF, iCAF, apCAF, and meCAF) have recently been identified, the epigenetic mechanisms that establish and maintain these subtypes remain poorly understood, representing an important area for future investigation. Furthermore, cell‐specific drug delivery systems may enhance therapeutic precision by enabling more effective targeting of distinct cellular populations within the TME while minimizing side effects.

## Conflict of interest

The authors declare no conflict of interest.

## Author contributions

JHP contributed to writing the original draft, review and editing, and visualization. M‐YK contributed to conceptualization, writing the original draft, supervision, and funding acquisition.
